# The mechanism and tumor inhibitory study of *Lagopsis supine* ethanol extract on colorectal cancer in nude mice

**DOI:** 10.1186/s12906-019-2585-6

**Published:** 2019-07-12

**Authors:** Lijuan Wei, Zhaoyong Wang, Yang Xia, Baichun Liu

**Affiliations:** 1 Department of Gastroenterology and Digestive Endoscopy Center, The Second Hospital of Jilin University, Changchun, 130041 Jilin China; 2 Department of Pathology, The Second Hospital of Jilin University, Changchun, 130041 Jilin China

**Keywords:** *Lagopsis supine* ethanol extract, Colorectal carcinoma, JAK/STAT signal pathway, Tumor xenografts

## Abstract

**Background:**

This study was aimed to determination the tumor inhibitory effect and explore the potential mechanisms of *Lagopsis supine* ethanol extract (Ls) on colorectal cancer.

**Methods:**

The cell growth inhibition experiment of Ls in colorectal cancer cell lines was determined by MTT method in the time course of 24, 48 and 72 h in four gradient drug concentrations. The protein expression levels of pSTAT3, pJAK2, STAT3, JAK2, Bcl-2 and caspase 3 were measured by Western blot method. The mRNA levels of the downstream genes of STAT3 were detected through semi-quantitative RT PCR. Sixty Balb/c-nude mice were xenograft with HCT116 colorectal cancer cells through subcutaneously. The xenografts were divided into five groups: model group, positive group (capecitabine 300 mg/kg) and three dosages of Ls treated groups (75, 150 and 300 mg/kg). Tumor size and tumor weight were calculated for evaluation the anti-tumor effects. H & E staining and immunohistochemical analysis were used to determine the histopathological changes and the levels of pSTAT3 and pJAK2 in the tumor tissues.

**Results:**

Ls exhibited a significant anti-proliferation effect in HCT116 and SW480 cells in vitro. The protein levels of pSTAT3, pJAK2 and Bcl-2, and the mRNA levels of Bcl-2 and Bak notably reduced with a dose-dependent manner. While the protein levels of caspase 3, and mRNA levels of Bax and caspase-3 remarkably increased in the gradient dosage of Ls in HCT116 cells. HCT116 in vivo xenografts experiment showed that the growth of the tumors significantly inhibited by Ls administration, which with no any significant body weight changes in each experiment group. The histopathology analysis displayed that Ls significantly reduced the inflammatory cells in tumor tissue. Furthermore, Ls also significantly down-regulate the protein levels of pSTAT3 and pJAK2 in the tumor tissues, compared with the model group.

**Conclusions:**

This work shows that Ls inhibited the cell proliferation of colorectal cancer in vitro and significantly reduced the tumor growth in HCT116 xenografts in vivo, which is probably related with the JAK/STAT signal pathway.

## Background

Colorectal cancer (CRC) is a common digestive tract malignancy, occurring in the colon and rectum. It is accounting for the second place in gastrointestinal cancer, and taking the third place in the incidence of cancer followed the lung cancer and gastric cancer, which generally observed after 40 years of age with a ratio of men and women as 2:1, serious threaten the human life and health in the worldwide [1–4]. Primary therapies applied to cure CRC are surgery, radiotherapy and conventional chemotherapy, despite progress in cancer control strategies of CRC, the patients survival rate have not been changed, especially for the patients with liver and lung metastases [5–8]. In recent years, with the changes of people’s living habits and diet structure, the incidence of CRC is increasing year by year in worldwide [9], treatment with one therapy method always could not achieve ideally efficacy [10, 11]. Currently, the drugs used for colorectal cancer in clinical therapy mainly include 5-fluorouracil (5-FU), oxaliplatin and doxorubicin, which all are chemotherapy reagents [12–14]. In addition, bevacizumab, cetuximab and panitumumab which are monoclonal antibodies designed based on specific targets [15–17]. However, these conventional anti-cancer drugs always have a high cytotoxicity effects, until now there is no any therapy method could totally rescue the people’s life in lower pains.

Traditional Chinese medicine (TCM) is one of the useful medical healthcare systems in China and other Asian countries, which has been focused by more and more medical researchers. Newman et al. made a statistics from 1981 to 2014, most of the approved cancer therapy drugs are derivatives of natural products [18, 19]. Due to the potential of multiple ingredients, multi-targets, multi-links and multi-pathways of the nature products, more and more people realized the importance of “return to nature”. Exploring the anti-tumor botanical compounds has gradually become the hot point for clinical researches on anti-tumor drugs [20–22]. At present, the more prominent multi-target anti-cancer drugs on the market such as sorafenib, dashatinib, sunitinib, lapatinib and so on. *Lagopsis supine* (Ls, Chinese name as Xiazhicao) has been used as a traditional medicinal herb for centuries in China, it is the dried whole plant of *Lagopsis supine* (Steph) IK. Gal., belongs to the Labiate family [23]. As a famous herbal medicine in TCM, it is described in the authoritative medical book of ancient China “Shennong’s Herbal Classics (Shen Nong Ben Cao Jing)”.

The chemical constitute study reported that Ls contains many bioactive components, such as diterpenoids, triterpenoids, steroids and saponins, flavonoids and flavonoid glycosides, phenylpropanoid glycosides, phenyl ethanol and alkaloids [24–26]. Modern pharmacological investigation reported that the crude extract of Ls have an improvement in myocardioprotective, anti-inflammation, anti-oxidative and anti-hepatic cancers [27]. Although the pharmacological research of Ls was more, the anti-tumor activity of Ls on colorectal cancers have not yet been studied so far. Therefore, we would like to study that if Ls has an anti-tumor effect on colorectal cancers. Moreover, if it has an activity, what the mechanism action is? Here, we will try our best to figure out the activity and action mechanism of Ls in the inhibition of colorectal cancers.

Above all, in this study, we would like to explore the mechanism of Ls on the inhibition of cell proliferation and tumor growth of colorectal cancer in vitro and in vivo, and provide more experimental/theoretical evidence for further elucidating the anti-tumor mechanism of Ls.

## Methods

### Materials and reagents

Human colorectal cancer cell line HCT116 and SW480 were obtained from American type culture collection (ATCC, Manassas, VA, USA). Ls (C1386-10 g) were purchased from Sigma. Capecitabine was obtained from Shang Hai Haoran Biological Technology Co. Ltd. (Shanghai, China). 3-(4,5-dimethyl-2-thiazolyl)-2,5-diphenyl − 2-H-tetrazolium bromide (MTT, NO. M8180) was obtained from Beijing solarbio science and technology Co. Ltd. (Beijing, China). Rabbit anti-human JAK2 (D2E12, #3230), pSTAT3 (Tyr705, D3A7, #9145), Mouse anti-human STAT3 (124H6, #9139), monoclonal antibody was purchased from Cell signaling Technology (CST). Goat anti-human pJAK2 (Tyr1007/Tyr1008, #sc-21870) was purchased from Santa Cruz Biotechnology. Mouse anti-human beta-actin monoclonal antibody was purchased from pocky Biotechnology (Suzhou) Co. Ltd..

### Cell culture

HCT116 and SW480 colorectal cancer cells were cultured in the medium of Dulbecco’s modified eagle medium (DMEM) with 10% fetal bovine serum (FBS) and 1% penicillin-streptomycin according to the conditions showed on American type culture collection (ATCC, Manassas, VA, USA). The cells were passaged in every 3 days and cultured in the 37 °C humidification incubator with 5% CO_2_. The phase of logarithmic cells was digested by 0.25% trypsin, cell numbers were counted and adjusted to 1 × 10^7^/mL by vaccination for experiment use.

### Cell proliferation assay by MTT method

The anti-proliferation activity of Ls in HCT116 and SW480 cells were measured by MTT (3-(4,5-dimethyl-2-thiazolyl)-2,5-diphenyl-2-H-tetrazolium bromide) method. The logarithmic growth phase cells of HCT116 and SW480 were planted in a 96-well plates with 5000 cells/well, respectively. Different concentrations of drug diluted with cell culture media, and added to the cells. The cell was continue incubated at 37 °C for 24 h, 48 h and 72 h respectively. Add 20 μL MTT solution to each well in the indicated times and incubate for 4 h. Discard the MTT supernatants carefully after MTT incubation, then add 100 μL DMSO to each well, shake plates for 10 min on a plate shaker by slowly increasing the shaking speed to a maximum of 900 shakes/min, to make sure the formazan crystals were thoroughly solved, then the absorbance of the plate were measured at a wavelength of 570 nm by a microplate reader. Each test concentration was performed in triplicates and repeated three times.

### Animals

Sixty male Balb/c-nude mice (body weight 18–20 g) were supported by the experimental animal center of the Jilin University (Changchun, China). The animals were reared in SPF grade animal rooms with the temperature of 24 ± 1 °C and the humidity of 50% ± 5% followed by 12 h day/night cycles. All the mice were free access to food and water, and quarantined for one week before animal experiment. The mice were received humane care and six mice raised in one polyacrylic cage in the terms of National Institutes of Health Guidelines of the USA (National Research Council of USA, 1996) and the University ethical regulations of Jilin University.

### Experimental design

The colorectal cancer cells of HCT116 were digested and adjusted to 1 × 10^7^/mL, the counted cells in cell medium were mixed with matrigel with the ratio of 1:1 on ice, and then take 200 μL of the cell mixture seeded into the right axilla in Balb/c-nude mice with the cell numbers of 1 × 10^6^/mice. The xenograft mice were randomly divided into five groups (*n* = 12): model group, Capecitabine (Cap) 300 mg/kg group [28], Ls 75 mg/kg group, Ls 150 mg/kg group and Ls 300 mg/kg group, when the volume of the tumor growth to 100 mm^3^, which need about two weeks from the initial inoculation. The selected dosage of Ls was calculated from human to mice according to Chinese pharmacopoeia of 2010.

Model group administrated with drug solvent (5% DMSO + 10% Tween 80 + 85% PBS) in 20 mL/kg body weight (BW) by oral administration once a day. The Cap and Ls groups orally administrated with 300 mg/kg of Cap and 75 mg/kg, 150 mg/kg, 300 mg/kg of Ls on the oral volume of 20 mL/kg BW, respectively. All the animals were sacrificed by cervical dislocation in the 15th days, after drug administration at the end of the experiment. The tumor tissues of all the xenografts were harvested and weighted. All the tumor tissue was fixed in 4% formaldehyde solution rapidly for 12–24 h, and embedded into paraffin for histopathological and immunohistochemically analysis.

### Western blot

The protein levels of pSTAT3, pJAK2, STAT3 and JAK2 after Ls treated in HCT116 cells were detected by immunoblot method. Before collection, the cells were washed with pre-cold PBS solution twice. The cells were collected with the scraper in RIPA (Radio Immunoprecipitation Assay, Beyotime Biotechnology, China) lysis buffer, collected into 1.5 mL tube on ice for 20 min, vortex twice. Cell lysate were centrifuged at 3000 rpm, 10 min, 4 °C, then collect the lysate and measure the protein concentrations with BCA kit (Solarbio, Beijing, China). Then add 5 × SDS loading buffer, 95 °C boiling for 10 min, stored at − 20 °C for western blot analysis.

### Quantitative RT PCR

Total RNA samples of different Ls treated HCT116 cells were extracted by Trizol kit (ZN00801, shinegene, Shanghai, China) under the manufacturer’s introduction. The model cDNA was got from the reverse transcription of total RNA (2 μg), according to PrimeScript®RT reagent kit, respectively. The mRNA expression levels quantified by RT-PCR. The sequences of all the primer were Bcl-2 (746 bp): forward 5′-GCTCTGAACAGATCATGAAGACAG-3′; reverse 5′-CAATCCAAAGTGGACC TGAGG-3′; Bax (169 bp): forward 5′-TGCTTCAGGGTTTCATCCAG-3′, reverse 5′-GGCGGCAATCATCCTCTG-3′; Bak (588 bp): forward 5′-GGATTGG TGGGTCTATGTTC-3′, reverse 5′-CTACACCCCTGGATTA CACTG-3′; Caspase-3 (683 bp) forward 5′-CTCGGTCTGGTACAGATGTCG ATG-3′; reverse 5′-GGTTA ACCCGGGTAAGAATGTGCA-3′; *β*-actin (200 bp) forward 5′-CTACAATGAG CTGCGTGTGG-3′; reverse 5′-TAGCTCTTCTCCAGGGAGG A-3′. The gene of *β*-actin was used as an internal control, in order to normalize the gene expression levels in each sample. The changes of the mRNA expression in all the groups were calculated by the method of 2-ΔΔCt [29].

### Histopathological analysis

Take 5 mm × 5 mm tumor tissues to 4% Formaldehyde solution for 12–24 h. Then embedded the fixed tissue to paraffin, and cut into 5 μm thickness sections. The sections stained with H&E according to the routine of the histopathological examination. Finally, the H&E stained sections were imaged and analyzed the degree of the inflammation under a light microscope at 200× magnification (BX-50 Olympus).

### Immunohistochemistry assay

Immunohistochemistry method [30] was used for the detection of the protein expression of pSTAT3 and pJAK2 in tumor tissue in each group. The EnVision two-step method was used in the process of the immunohistochemically experiment. The kit was obtained from Beijing Zhongshan biotechnology company (Beijing, China). The paraffin-embedded sections were cut into 5 μm thickness sections, and the sections were mounted on the glass slides, then deparaffinized, quenched and incubated with the primary antibody for 12 h, respectively. After the primary antibody incubation, the sections blocked with goat serum for 20 min. Then the sections incubated with the second HRP-conjugated goat anti-mouse antibody for 30 min at 37 °C, respectively. The final positive signals visualized by DAB-H_2_O_2_ for 10 min at room temperature. Images were magnificated of 200× (Olympus BX-50 Microscope and a Leica DMI; Leica Microsystems).

### Statistical analysis

The values presented in the study were represented as mean ± SD. One-way ANOVA and Repeated measures ANOVA test followed by Dunett’s t-test was used as a calculated statistical method with Statistical Product and Service Solutions (SPSS) 19.0 statistical software. *P* < 0.05 and < 0.01 were regarded as statistically significant.

## Results

### Cell growth inhibitory effect of ls on colorectal cancer cells

Before the in vivo anti-tumor experiment, we first detected the inhibitory effects of Ls on HCT116 and SW480 colorectal cancer cell lines in vitro. From the data of Table 1, we could see that the cell growth inhibitory effect of Ls was remarkably increased in the dosage of 30, 60, 80 and 100 μM in HCT116 and SW480 cells (*P* < 0.05, *P* < 0.01), compared with the normal group. In addition, the cell growth inhibitory effects significantly increased in the longer of the treatment times (24, 48 and 72 h, *P* < 0.05, *P* < 0.01). Furthermore, from the result, we can see that the cell growth inhibitory effect is slightly sensitive in HCT116 cells than SW480 cells during the treatment times of 24, 48 and 72 h (Table 1).

**Table 1:** Anti-proliferation effect of Ls on HCT116 and SW480 colorectal cancer cells

Group (μM)	Inhibitory ratio% (HCT116)				Inhibitory ratio% (SW480)			
	24 h	48 h	72 h	F	24 h	48 h	72 h	F
Control	0.0	0.0	0.0		0.0	0.0	0.0	
Ls 30	18.7 ± 1.2^*^	38.4 ± 2.2^*#^	51.1 ± 1.9^*##^	4586.146	23.6 ± 0.6^*^	31.0 ± 0.6^*^	46.4 ± 1.7^*#^	277.732
Ls 60	35.2 ± 3.0^**^	50.5 ± 1.7^**#^	69.3 ± 1.6^**##^	217.559	30.7 ± 0.8^**^	39.8 ± 1.5^**^	51.8 ± 1.5^**#^	111.204
Ls 80	70.8 ± 3.3^**^	86.1 ± 2.9^**#^	90.7 ± 2.7^**#^	16.067	50.5 ± 1.1^**^	69.9 ± 1.7^**#^	87.2 ± 3.1^**##^	107.379
Ls 100	89.6 ± 4.0^**^	92.7 ± 5.1^**^	98.3 ± 4.4^**#^	92.333	74.8 ± 2.2^**^	88.3 ± 2.1^**#^	90.5 ± 2.4^**#^	154.151

Data are expressed as mean ± SD for each group. The data was repeated for three independent times. ^*^P < 0.05, ^**^P < 0.01 vs control group. ^#^*P* < 0.05, ^##^*P* < 0.01 vs 24 h treatment data. Ls: *Lagopsis supine* ethanol extract

### Effects of ls on protein expression of *p*STAT3, STAT3, *p*JAK2 and JAK2 in HCT116 cells

**Fig. 1:**
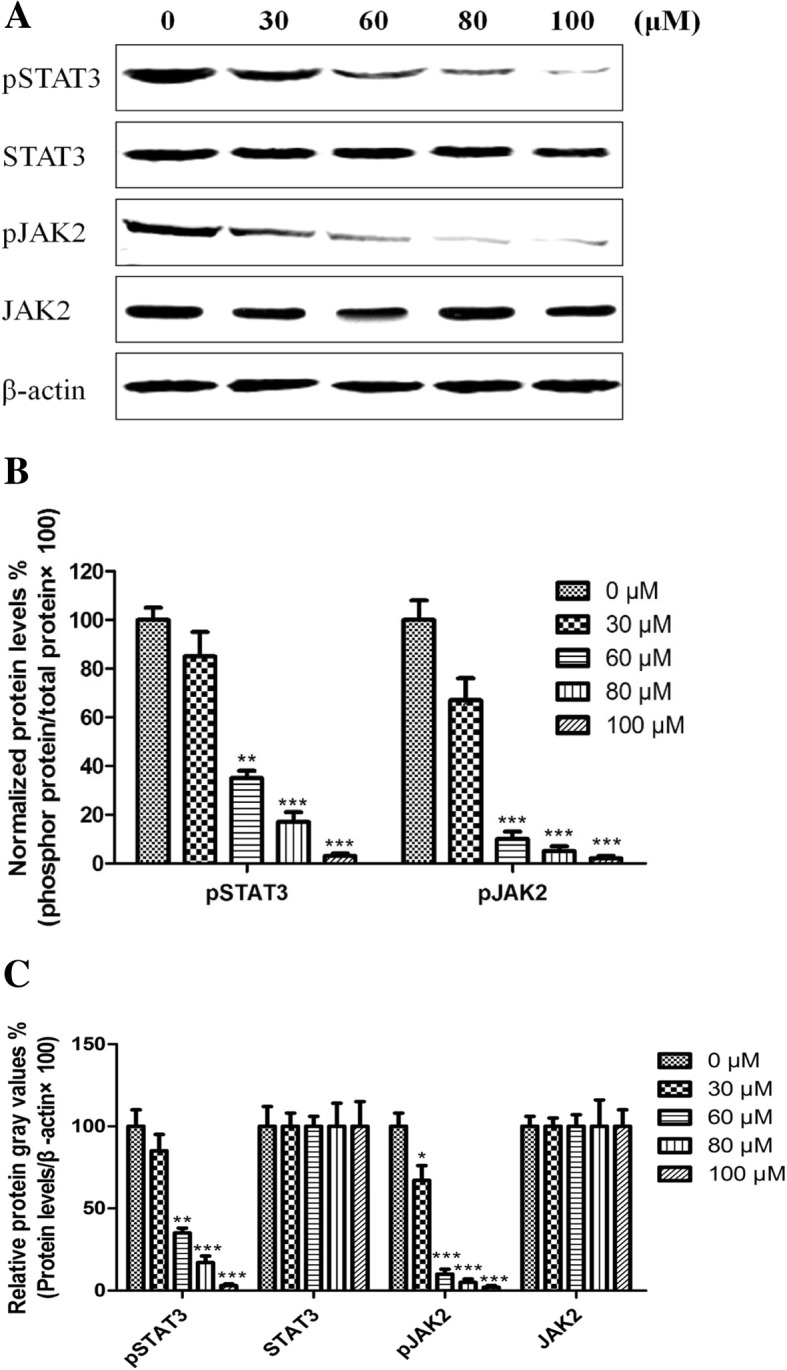
Effects of Ls on protein expression of *p*STAT3, STAT3, *p*JAK2 and JAK2 in HCT116 cells. (**a**) The represented western blot images for all the groups. (**b**) Normalized phosphorylation levels of STAT3 and JAK2 to the total relevant proteins (STAT3 and JAK2 respectively). (**c**) The statistical data for the western blot results of all the groups in three independent times. ^**^P < 0.01, ^***^P < 0.001 vs control group

Under the above result of the in vitro cell growth inhibitory effect, we planned to study if Ls has an effect on JAK/STAT signal pathway. Here, we choose colon cancer HCT116 cells as the object. Data from one representative experiment demonstrated that treatment with 30, 60, 80 and 100 μM of Ls obviously decreased the phosphorylation levels of STAT3 and JAK2 compared to untreated cells (Fig. 1a). Then the phosphorylation levels of STAT3 and JAK2 were normalized to the total relevant proteins (STAT3 and JAK2 respectively, Fig. 1b) and accumulated data from three independent experiments revealed that treatment of HCT116 cells with different concentration of Ls significantly decreased the phosphorylation levels of STAT3 and JAK2 (Fig. 1c), which showed a clearly dose-dependent manner. The result exhibited that the cell growth inhibitory effects of Ls maybe related with the signaling pathway of JAK/STAT.

### Effects of ls on mRNA and protein expression of Bcl-2, Bax, Bak and Caspase-3

**Fig. 2:**
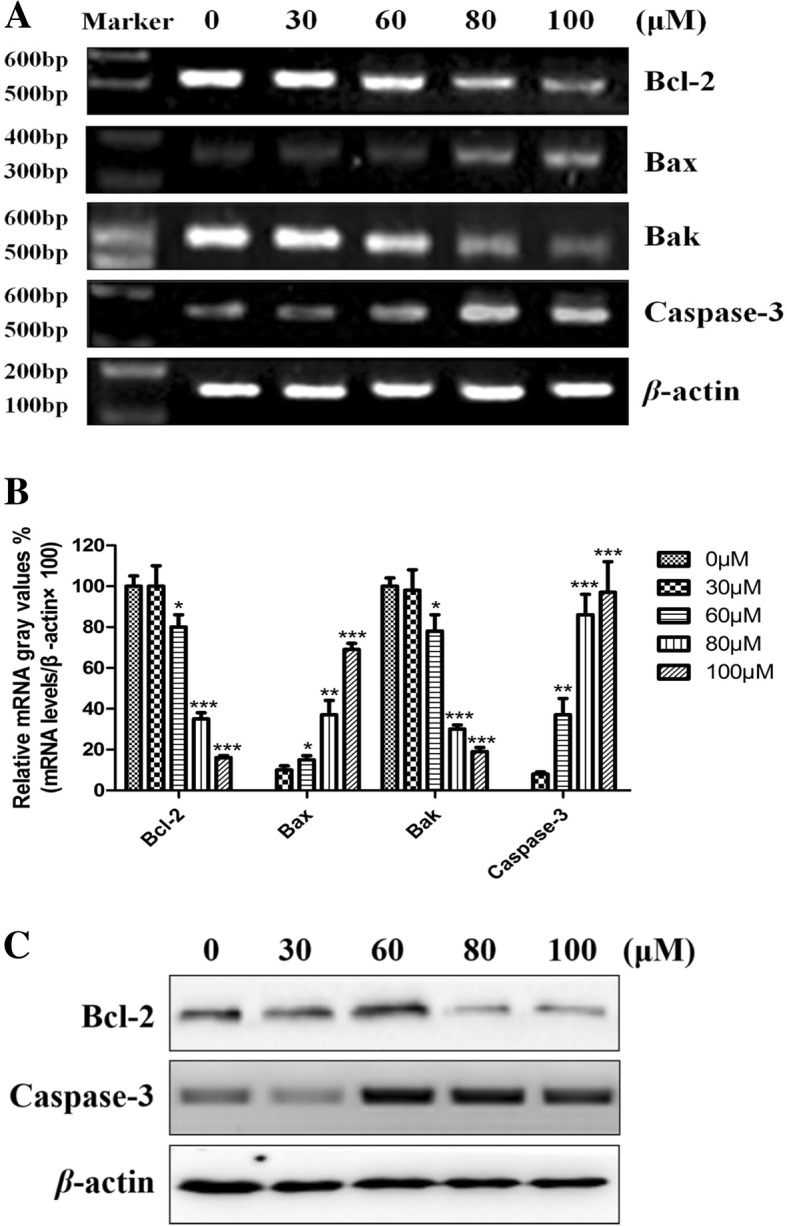
Effects of Ls on the expression of Bcl-2, Bax, Caspase-3 and Bak genes in HCT116 cells. (**a**) The represented images for mRNA levels in all the groups. (**b**) The statistical data for the mRNA levels in all the groups. (**c**) The represented images for protein expression levels of Bcl-2 and Caspase-3 in all the groups. The data was repeated in three independent times. ^*^P < 0.05, ^**^P < 0.01, ^***^P < 0.001 vs control group

Furthermore, we also detected the apoptosis related genes expression (Bcl-2, Bax, Bak and Caspase-3), which showed in Fig. 2. As the data of the Fig. 2, we can see that treatment with Ls significantly decreased the mRNA expression of Bcl-2 and Bak, especially in the group of 100 μM Ls. While the mRNA expression of Bax and Caspase-3 markedly increased in the dosage of 30 to 100 μM with a dose-dependent manner, compared to the control group (Fig. 2a,b). In addition, the Western blot assay showed that Ls significantly increased the protein expression of caspase 3, and decreased the protein expression of Bcl-2, which consistent with the mRNA levels (Fig. 2c).

### Effect of ls on HCT116 tumor xenografts

**Fig. 3:**
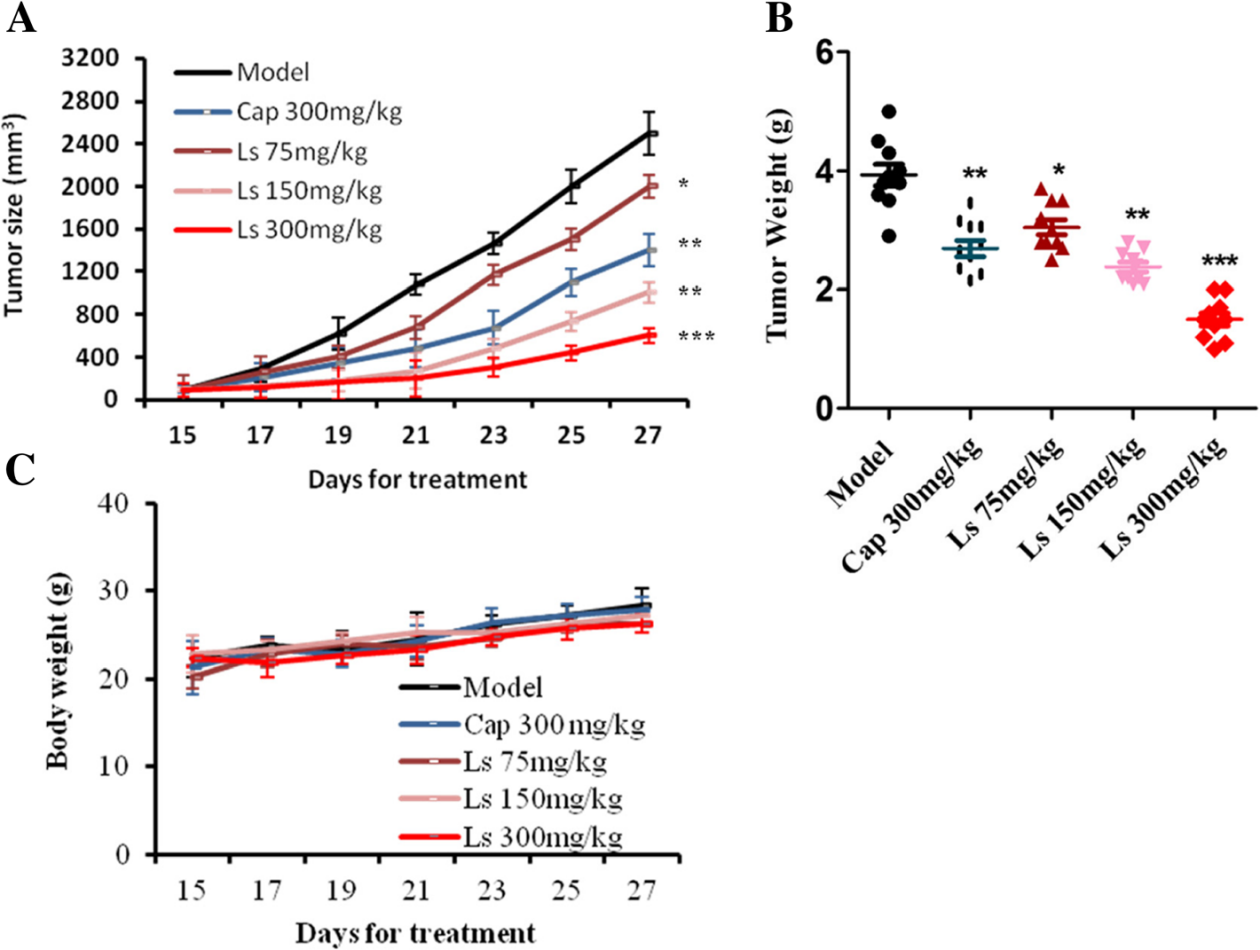
Effects of Ls on HCT116 tumor xenografts (*n* = 12). (**a**) tumor size, (**b**) tumor weight and (**c**) body weight. ^*^P < 0.05, ^**^P < 0.01, ^***^P < 0.001 vs model group

In the following experiment, we studied the in vivo anti-tumor effect of Ls on HCT116 tumor xenografts through subcutaneous injection. As the result of Fig. 3, it is showed that Ls displayed a remarkable tumor inhibitory effect on HCT116 xenografts (*P* < 0.05, *P* < 0.01), especially the dosage of 300 mg/kg, compared to the model group. Figure 3a showed the tumor size in different groups, it exhibited that Ls significantly reduced the tumor size in all the drug treatment groups (75, 150 and 300 mg/kg, *P* < 0.05, *P* < 0.01). While the final tumor weight in all drug treated groups were also decrease significantly, compare with the model group (Fig. 3b, *P* < 0.05, *P* < 0.01). Furthermore, we did not observe any changes in the animal body weight in the whole progress of the experiment, and all the animals were survived in the whole experiment (Fig. 3c), which reflected that Ls has a very lower side effects in vivo experiment.

### Histopathological changes in the tumor tissue

**Fig. 4:**
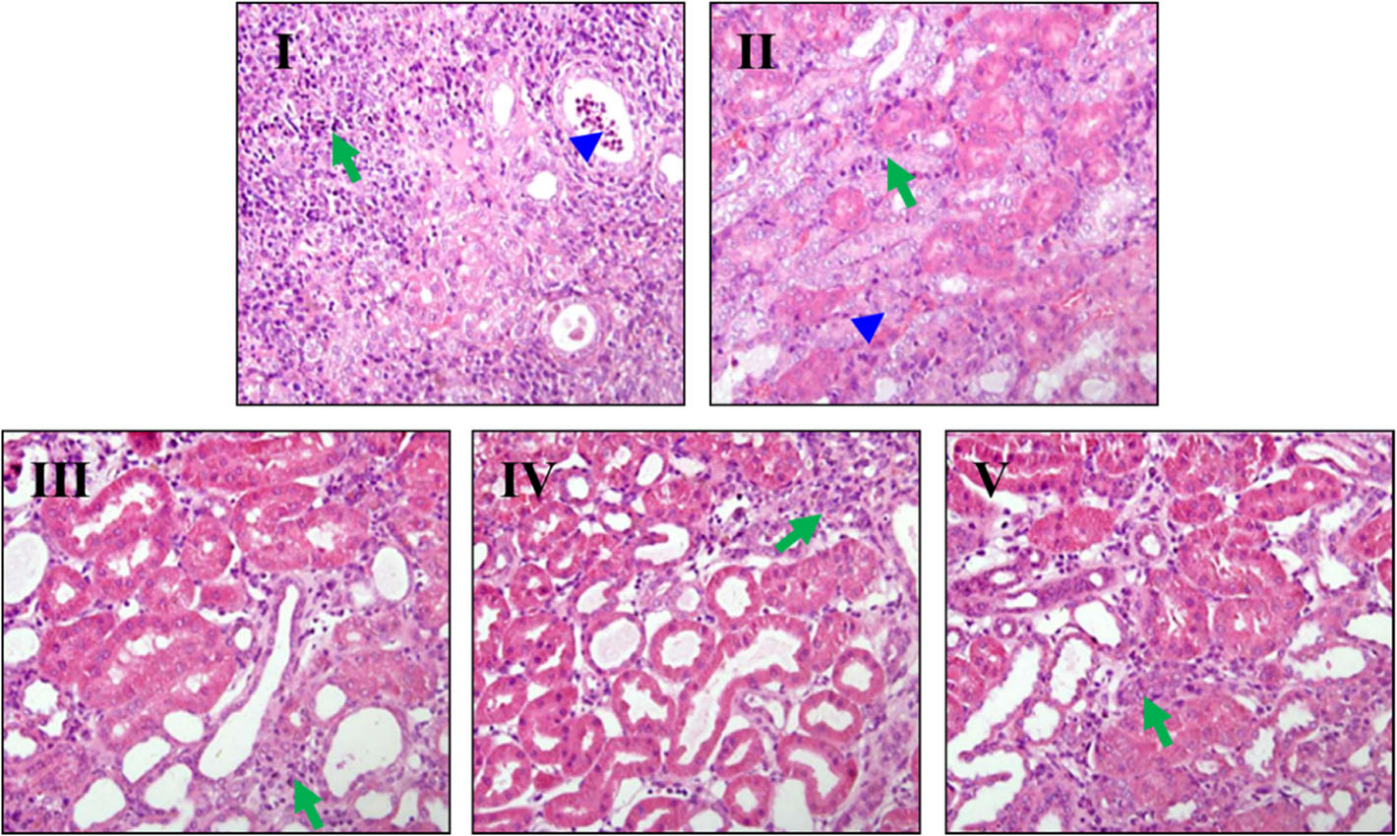
Histophathological changes after Ls treatment in HCT116 xenografts by H&E staining (200×). I: Model group, II: Cap 300 mg/kg, III: Ls 75 mg/kg group, IV: Ls 150 mg/kg group, V: Ls 300 mg/kg group. The retention of intra-cavitary mucus marked in blue arrowhead, the peripheral lymphocyte infiltrations marked in green arrows in the pictures

In order to observe the histology changes of the xenografts after Ls treatment, we continue analyzed the pathological changes in tumor tissue through H&E staining in all the experiment groups. From the photomicroscope image in Fig. 4, the tumor in the model group showed that there exist many gland cancer nests, lots of intra-cavitary mucus retention (marked in blue arrowhead), the nuclear size was all no uniformitarian, and there have many peripheral lymphocyte infiltrations (marked in green arrows) in the tumor tissue (Fig. 4 I). While in the group of 75 mg/kg, the tumor tissue showed less mucus retention and visible peripheral lymphocyte infiltration (Fig. 4 III). In the tumor tissue of the group of 150 mg/kg, the visible acute inflammatory cells were infiltration in tumor and peripheral tissues (Fig. 4 IV). Furthermore, the tumor tissue in the dosage of 300 mg/kg group showed a decreased secretary cells and acute inflammatory cell infiltration in tumor and peripheral tissue, the tumor tissue was markedly degenerated (Fig. 4 V).

### Effect of ls on *p*JAK2 and *p*STAT3 levels in HCT116 xenografts by IHC analysis

**Fig. 5:**
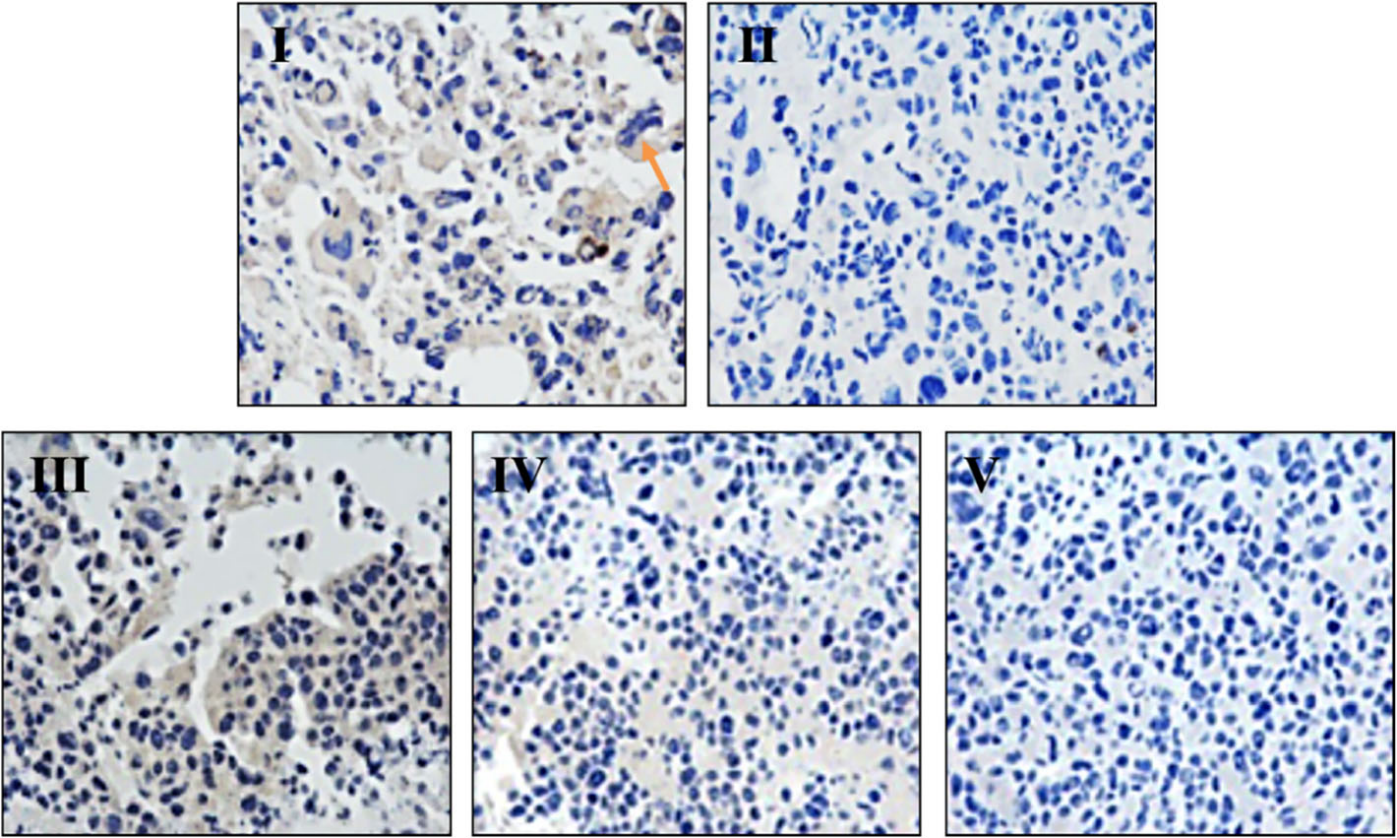
Representative IHC microphotographs of *p*JAK2 in HCT116 xenografts (400×). I: Model group, II: Cap 300 mg/kg, III: Ls 75 mg/kg group, IV: Ls 150 mg/kg group, V: Ls 300 mg/kg group. The protein expression of *p*JAK2 displayed as brown, mainly located in cell cytoplasm, which marked with the yellow arrow in the pictures

**Fig. 6:**
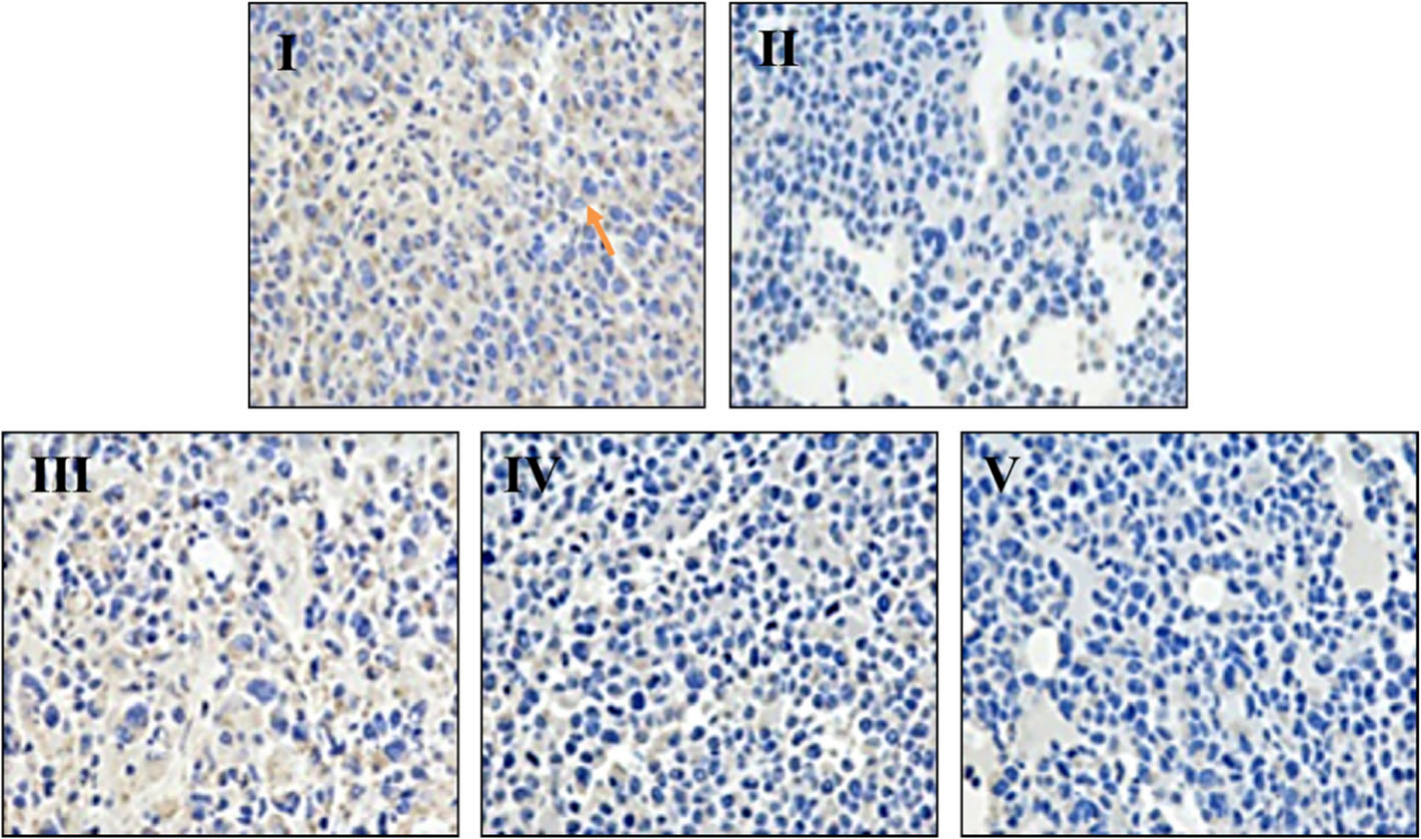
Representative IHC microphotographs of *p*STAT3 in HCT116 xenografts (400×). I: Model group, II: Cap 300 mg/kg, III: Ls 75 mg/kg group, IV: Ls 150 mg/kg group, V: Ls 300 mg/kg group. The protein expression of *p*STAT3 displayed as brown, mainly located in cell cytoplasm, which marked with the yellow arrow in the pictures

To detect the related mechanism of Ls in vivo, we analyzed the protein expression of *p*JAK2 and *p*STAT3 in Ls treated tumor tissue (Fig. 5 and Fig. 6). The positive protein expression of *p*JAK2 and *p*STAT3 displayed as brown in tumor tissue, which mainly existed in cell cytoplasm, and marked with the yellow arrow (Fig. 5 and Fig. 6). In model group, the protein expression of *p*JAK2 and *pSTAT3* was obviously increased in cell cytoplasm (Fig. 5 I and 6 I). After Ls treatment, the protein expression of *p*JAK2 and *p*STAT3 were significantly decreased in all the Ls treated groups (75, 150 and 300 mg/kg), which displayed with a dose dependent manner (Fig. 5 III-V and 6 III-V).

## Discussion

*Lagopsis supine* (Steph) IK. Gal. (Ls), a famous herbal in TCM, is widely distributed in China. Recent researches have reported that it has many bioactive constitutes, and possess many pharmalogical activities in the anti-oxidant, anti-inflammation and anti-cancers [27]. In this study, we mainly studied the effects of Ls on colorectal cancers in vitro and in vivo, clarified its effect on the signaling pathway of JAK/STAT, and further discussed the possible mechanism of Ls for colorectal cancer.

STAT3, a tyrosine phosphorylation of signal channel coupling bifunctional protein, exist in cell cytoplasm, plays an important role in transmitting signals [31]. It has been found that a variety of extracellular signals can activate STAT3, then forming a two dimers and come into the nucleus, to recognize and bind to specific DNA promoter, in order to regulate the transcriptional of the target gene [32, 33]. STAT3 signal transduction pathway is activated in a variety of primary tumors and tumor derived cell lines [34], which plays an important role in the regulation of tumor cell growth, proliferation and apoptosis process, this is may be related to the activation of a series of target gene followed STAT3 activation [35, 36]. In the recent years, there are many researches on the relationship between STAT3 and cancers, and these research suggest that the activation of STAT3 is closely associated with colorectal cancer [37]. Thus, STAT3 as a therapeutic target for colorectal cancer may provide a new therapeutic strategy.

Apoptosis is an important process during tumor regression. The Bcl-2 family is the regulatory center of mitochondrial pathway to induce apoptosis. The apoptosis suppressor gene Bcl-2 and the apoptosis promoting gene Bax form heterologous two dimers. The ratio between them is related to the regulation of apoptosis, which directly determines the apoptosis of cells [38]. Caspases, a cysteine protease family, is the key factor for the apoptosis process, it can activate the whole protease family after itself activation [39, 40]. Therefore, once the apoptotic process is triggered, it will become a caspases cascade reaction. While among the proteins of the caspase family, caspases-3 is an important apoptosis effector, it can be activated by the upstream initiating subsystem, and effect to the specific substrate to make the cell biochemical changes and morphological changes, and then finally lead to cell apoptosis.

In the study, our results showed that Ls can effectively inhibit the cells proliferation in human colorectal cancer cell line HCT116 and SW480 with the increased the drug concentration and the prolongation of treated times, the inhibitory effect was displayed a dose and time dependent manners. The results of this study also showed that with the increased concentration of Ls, the mRNA expression of Bcl-2 and Bak were significantly decreased, and the mRNA expression of Bax and caspase-3 were remarkably increased, which suggest that Ls caused the cancer cells apoptosis. Furthermore, with the increased treated concentrations of Ls, there showed a less or not significant changes in the protein expression of STAT3 and JAK2, while the protein expression levels of *p*STAT3 and *p*JAK2 were gradually decreased, which reflected that Ls could inhibit the activation of *p*STAT3 and *p*JAK2 in colorectal cancer cells. In addition, the in vivo xenografts experiment displayed that treated with Ls significantly decreased the tumor size, especially in the dosage of 300 mg/kg. The IHC analysis exhibited that Ls markedly reduced the protein expression of *p*STAT3 and *p*JAK2 in tumor tissue. These experimental results indicating that Ls inhibit the activation of downstream target genes may through inhibiting the activation of STAT3, which continue weakening its inhibitory effect on tumor cell apoptosis and further promoting the apoptosis of colorectal cancer cells.

## Conclusion

This study explored the effect and the mechanism of Ls in colorectal cancer. The results showed that Ls could significantly inhibit the growth and proliferation of colorectal cancer cell line HCT116 and SW480 with a dose dependent manner under the concentration range of 30–100 μM. The in vivo xenograft experiment showed that Ls significantly inhibited the tumor growth, which may be through down-regulation the protein expression of Bcl-2 and Bak, up-regulation the protein expression of Bax and Caspase-3. In addition, the result also showed that Ls could block the activation of STAT3 in human colorectal cancer HCT116 cells, which maybe the mechanism of Ls in anti-tumor effect.

## Data Availability

The datasets used and/or analysed during the current study available from the corresponding author on reasonable request.

## Abbreviations

5-FU: 5-fluorouracil
BW: Body weight
Cap: Capecitabine
CRC: Colorectal cancer
DMEM: Dulbecco’s modified eagle medium
FBS: Fetal bovine serum
H&E: Hematoxylin and eosin
IHC: Immunohistology chemistry
Ls: *Lagopsis supine*
TS: Tumor size
TW: Tumor weight
